# Module Parasitics-Based Current and Temperature Sensing Using Explainable Neural Networks

**DOI:** 10.3390/s26072235

**Published:** 2026-04-04

**Authors:** Frank Lautner, Mark-M. Bakran

**Affiliations:** Department of Mechatronics, Centre for Energy Technology, University of Bayreuth, 95447 Bayreuth, Germany; bakran@uni-bayreuth.de

**Keywords:** current sensing, junction temperature estimation, power electronics module, artificial intelligence, artificial neural networks, explainable AI

## Abstract

**Highlights:**

**What are the main findings?**

- Current sensing via parasitic sections of a power semiconductor module can be done with the help of neural networks to cut out side effects.
- These networks can easily be tested to determine whether they are physics-grounded or not.

**What are the implications of the main findings?**

- Expanding current sensing to include junction temperature estimation and additional input parameters is also possible; however, simple explainability tests often show a very selective behaviour towards high-correlation signals.
- Forcing the neural network to work with poorer-quality measurement data may lead to acceptable overall performance, but it shows some degree of specialisation instead of a generalised physics-grounded behaviour.

**Abstract:**

This paper examines the application of simple neural networks for current measurement and the determination of the junction temperature in power semiconductor modules. On the one hand, the focus was not on the use of conventional sensors such as current sensors or temperature sensors, but rather on utilising parasitic components within the power semiconductor module, from which useful signals can be extracted. Namely, these are the voltage across parasitic inductances in a module, the semiconductor’s on-state voltage, and its turn-on delay time. Because these signals are often affected by other parameters, the desired information must be extracted, which was found to be an application case for artificial neural networks. On the other hand, the application of ANNs in the simplest and most effective way possible was presented. Furthermore, a method is introduced that takes a first step towards the interpretability of neural networks in a straightforward manner to overcome the main drawback for the user—the usual black-box structure of neural networks.

## 1. Introduction

Power inverters are costly and maintenance-intensive parts of electric drivetrains and other applications that require electric motors. Because of that, it is interesting to take a closer look at the question of where possible savings (monetary and maybe in other aspects) can be achieved within these components. One obvious option is the sensing system of a power inverter. For an efficient and safe operation, a variety of parameters are relevant. Firstly, the current through the power electronic switch is of utmost importance for protection. Secondly, the inverter’s output current must be known precisely to ensure correct torque and magnetic flux in the motor. Another important parameter is the temperature of the power electronic switch. This value is an indication of the utilisation of the module and can be used for condition monitoring, which, in addition, needs other parameters like module temperature and coolant temperature. Other parameters like DC voltage are effortlessly obtainable but are also useful for a holistic monitoring of the power module. This work focuses on the main key figures, which are the current through a semiconductor switch (representing the inverter current) and the power switch’s temperature (and especially the junction temperature). Current sensing can be achieved using approaches that exploit, for example, the magnetic field of the flowing current—processed with Hall sensors [1], GMR sensors [2,3], and TMR sensors (including AI-assisted methods [4])—and via shunts [5]. Also, more indirect approaches have been discussed lately by exploiting module parasitics that must, in turn, deal with perturbations, for instance [6], where the parasitic resistance is mitigated rather than determined like in the proposed method. The use of neural networks has been discussed recently in the field of current sensing, often in combination with additional sensors, such as Hall-effect sensors described in [7]. Other applications in the field of current sensing and the simultaneous usage of AI also comprise indirect signals [8], though not specifically parasitic ones.

A similar situation arises when determining the junction temperature, which is another step towards condition monitoring. In this case, accurate measurement often requires additional sensors [9]. Measurement using inserted parts of the semiconductor is a similar approach [10]. Direct approaches reproduce the measured variable with high precision. However, they require additional, usually costly sensors or a significantly increased space within the power module, which must be avoided for efficiency reasons. Therefore, the focus should be on indirect methods. Indirect methods involve recording measured variables that are influenced by both the parameter of interest and significant lateral effects. These variables are known as temperature-sensitive electrical parameters (TSEPs) [11]. In this paper, both current sensing and junction temperature measurement are presented without additional sensors. Instead, available signals (TSEPs and ‘current-sensitive electrical parameters,’ CSEPs) are processed using artificial intelligence to reduce perturbations. Only recent studies have reported approaches with AI in these areas of research, like [12,13,14]. These studies often focus solely on the junction temperature and less on the current sensing. For example, ref. [15] uses the NTC-sensor of a power module; in [16], a variation in other operating parameters is tested instead. Another recent approach [17] uses waveforms during the turn-off of the switch and processes these signals using a convolutional neural network. There are a few references in the literature to systems based solely on indirect or parasitic signals, as such systems are prone to significant cross-talk or interference. When such methods are discussed, attempts are made to isolate the information content of the signals as much as possible using hardware techniques; for example, in [18], a voltage peak is used as current information by exploiting the parasitic components of a module.

Another aim of this paper is to present more than just neural network approaches for processing sensor data. In addition, it will also add ‘explainability’ to the usual resulting black-box models. The aim of this paper is not the consideration of single sensing methods (be it for current sensing or for temperature sensing) but the combination of different, rather low-cost sensing approaches, with simple neural networks. When taking such a holistic view, it is important to have a clear overview of the individual methods and the neural networks used. Therefore, this paper refrains from providing a detailed description of all the specifics of the methods used; instead, it aims first and foremost to provide an overview of the accuracies achievable with the sensor methods. Secondly, the neural networks used should be simple, feedforward networks that do not achieve excessive depth. Naturally, there will be more specialised methods for individual monitoring approaches that offer higher accuracy in specific cases. However, in such cases, the concept presented here—a combination of low-cost sensors and low-effort processing—would not be adhered to.

## 2. Proposed Current Sensing Technique

### 2.1. Parasitic-Based Current Sensing

**Figure 1:**
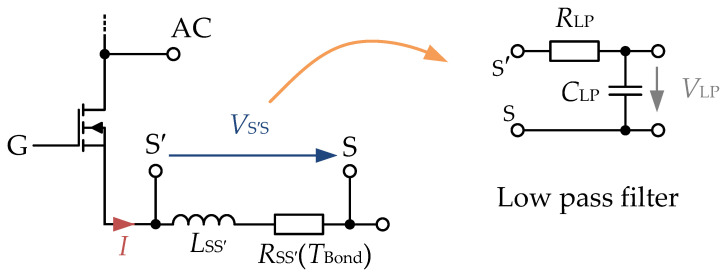
Current sensing scheme proposed in [20]. The current-sensitive signal *V*_S′S_ is processed with an integrating network to provide a current-proportional signal.

The principle of a current measurement via parasitic components in a power module can be seen schematically in Figure 1. Here, the voltage is measured at the taps of the source and the auxiliary source, for which these two connections must be available and accessible. Furthermore, it is necessary that there also be significant resistance and inductance between these two connections. However, all these conditions are met in many power semiconductor modules. This is especially the case when the load connection is also considered (e.g., [19] at least for some of the installed switches). Particularly when the investigated measurement section is very small and only a low parasitic inductance is present, it may be advisable to also consider the present, parasitic resistance, as indicated in Figure 1.

Current sensing via the parasitics of a power module is well known [21], but its use is limited in use (e.g., for overcurrent protection [22]) due to the difficulty of eliminating side influences. Therefore, this type of current measurement was usually only used for monitoring an overcurrent event. The advantage of such a procedure was above all the short reaction time that was made possible. As soon as current flows through the semiconductor (change in current flow), a voltage can be measured between the source and the auxiliary source (inductance voltage–current relationship) according to Equation (1).

(1) $$V_{S^{\prime }S}(t)=\frac{dI(t)}{dt}\cdot L_{{\mathrm{SS}}^{\prime }}+I(t)\cdot R_{{\mathrm{SS}}^{\prime \prime }}$$

To improve understanding, particularly in the subsequent description which extends this equation and incorporates signal processing, it is helpful to consider this signal in its Laplace-transformed representation. This eventually leads to Equation (2).

(2) $$V_{S^{\prime }S}(s)=s\cdot I\cdot L_{{\mathrm{SS}}^{\prime }}+I\cdot R_{{\mathrm{SS}}^{\prime }}$$

During switching operations or short-circuit events, a very high voltage can be measured for a short time, which can then ideally be processed directly via analogue networks. Any kind of interference would be unlikely to have a lasting effect on such a dominant signal.

If this signal, however, is used as an additional or stand-alone current sensing method, more precise processing of the measured signal is required. It is possible to use it without any processing of the $V_{S^{\prime }S}$-signal, but it is very difficult to handle because, in this case, the signal’s peak would need to be sampled very quickly and also accurately. Because of that, most approaches tend to process the raw signal with an integrator or at least a partly integrating network such as active low pass filters (the low pass filter part is also depicted in Figure 1). Neglecting the ‘active’ part—in other words, the proportional gain of the network—the transfer function $G_{\mathrm{LP}}(s)$ of such a filter can be described according to Equation (3).

(3) $$G_{\mathrm{LP}}(s)=\frac{\;1}{s\cdot \left(R_{\mathrm{LP}}\cdot C_{\mathrm{LP}}\right)+1}$$

In order to gain an ideal representation of the current according to Equation (4), the time constant formed by the values $R_{\mathrm{LP}}$ and $C_{\mathrm{LP}}$ of such a filter should match the time constant of the measurement section.

(4) VLPs=Is·s· LSS′RSS′+1s·RLP·CLP+1·RSS′

However, if this voltage signal has to serve as a measurement variable for the flowing current, secondary effects must certainly be considered. For example, in order to extract the current information from the tapped signal, it must be known exactly how the measurement path is configured and therefore what time constant is present.

This is challenging, especially when the parasitic resistance, which is temperature-dependent, has a significant influence on the measurement section time constant. For instance, at a higher temperature, the parasitic resistance between the source and the auxiliary source tap increases and provides a different signal than at lower temperatures. These deviations scale approximately with the temperature coefficient of the material used for the path between source and the auxiliary source (usually copper or aluminium, i.e., about 0.4%/K—meaning a 40% deviation at a temperature difference of 100 K). This problem would clearly make accurate current sensing impossible. In cases involving large parasitic inductance and comparatively low resistance, it may be possible to mitigate the temperature influence by sampling the value very shortly after the switching process. Assuming the measuring section consists of a series circuit with a small parasitic resistance and a dominant parasitic inductance, a simple integrator can be used to evaluate the signal. This would provide an accurate re-calculation of the current during the switching process and the phase in which the influence of the parasitic inductance, in combination with the high d*i*/d*t* value, predominates. After a certain time, only the current via the parasitic resistance will be measured at the tap of the measuring section. This is determined solely by the connected load and will only increase or decrease slightly (compared to switching the semiconductor). The integrator will then integrate this almost constant signal. This leads to a drift of the current measurement signal. Thus, if a current measurement was realised via a simple integrator, the time range between the decay of the switching disturbances and the beginning of the integrator’s drift behaviour would have to be very narrow. However, using an active low-pass filter with a certain time constant instead of an integrator requires accurate temperature compensation.

### 2.2. Temperature Compensation

Any additional form of temperature sensing (in order to compensate this parameter) is challenging, because common temperature sensing techniques in power semiconductors focus on the junction temperature of the chip. This temperature, however, is less useful for this problem, because the current sensing measurement section is located in the bond wires and conducting planes of the semiconductor module and thus shows a significantly different temperature [23]. This temperature may be affected by the current traversing the bond wire, or even by the temperature of the neighbouring switch because a cold adjacent part of the semiconductor will act as a better heatsink than a hot part and providing a certain potential for cooling through the bond wires. Therefore, it would be unreasonable to determine a specific bond wire temperature, as the overall resistance is made up of many different sections of the bond wire, each with its own respective temperature. Therefore, it is not really important to know the exact temperature of the bond wire at any point in the measurement section, but rather to have a good compensation possibility for the current signal.

This can be achieved as follows: If the integrator is tuned to one reference situation (i.e., a reference temperature) and the *LR*-measurement section is ideally compensated, the exact current waveform will be obtained at the integrator’s output. However, if another temperature is present, and the integrator has not been adjusted, a distorted signal will be seen at its output. This waveform can be described as a signal that is like the typical output of a PDT1-integrator (see Equation (4), whereby the factors $L_{{\mathrm{SS}}^{\prime }}/R_{{\mathrm{SS}}^{\prime }}$ and $R_{\mathrm{LP}}\cdot C_{\mathrm{LP}}$ do not match). In a conventional setting, this would be a significant disadvantage, because it would introduce a temperature-dependent nonlinear distortion to the measurement signal. The only solution to avoid such errors would be to wait a relatively long time after switching, so that only the proportional part of the integrator remains dominant. However, this would require a very stable op-amp device (if used). In the presented approach, however, the nonlinear change of the integrator signal itself is used for the purpose of temperature compensation, because the exact shape of the waveform is a characteristic of the temperature, as can be seen from a more precise formulation of Equation (4), viz. Equation (5).

(5) VLPs=Is·s· LSS′RSS′(TBond)+1s·RLP·CLP+1·RSS′(TBond)

Now, the waveform characteristics must be extracted in order to obtain the current information. This takes part in the time domain, which is why this equation may be transformed from its Laplace-form into time domain:

(6) VLPt = I^·RSS′(TBond)·1 + LSS′RSS′(TBond) − RLP·CLPRLP·CLP·e − tRLP·CLP

During the inverse transformation from the Laplace domain to the time domain, it was assumed that an ideal step of current with the magnitude $\hat{I}$ occurred at time *t* = 0. This fairly accurately reflects the situation during ‘switching on’ of the semiconductor, neglecting only the current overshoot, which should be much shorter than the time spans considered when using this method.

In order to obtain information from this signal, it must be sampled. This should be done as frequently as possible, but at least as often as is necessary to extract the desired information. If the signal is assumed to be influenced by the two parameters ‘current’ and ‘bond wire temperature’, two samples are necessary. In this case, a set of two equations containing two variables can be obtained. These consist of Equation (6), with *t* substituted by the sample times $t_{\mathrm{sample},1}$ and $t_{\mathrm{sample},2}$, respectively.

This set of equations must now be solved. Then, the current and even the temperature information can be determined for every switching process. This procedure and some results were presented in [24], where it was done mostly via conventional mathematics.

However, as discussed in the referenced publication, this problem can also be solved with the help of artificial intelligence. By recording enough training data, which consists of a pair of the variables $I_{D}$ and $T_{\mathrm{Bond}}$, and a pair of signal samples, a simple (i.e., shallow) artificial neural network (ANN) can be trained. This ANN must learn the correlations between the signal waveform and the underlying parameters present in the actual switching process. If a feedforward network is to be used, the relationship can (and will) be nonlinear, but it should not be time-dependent itself. This means that all switching processes should be independent of each other. In this case, networks that provide additional features are necessary. In this paper, however, the focus is on the common, independent switching processes.

**Figure 2:**
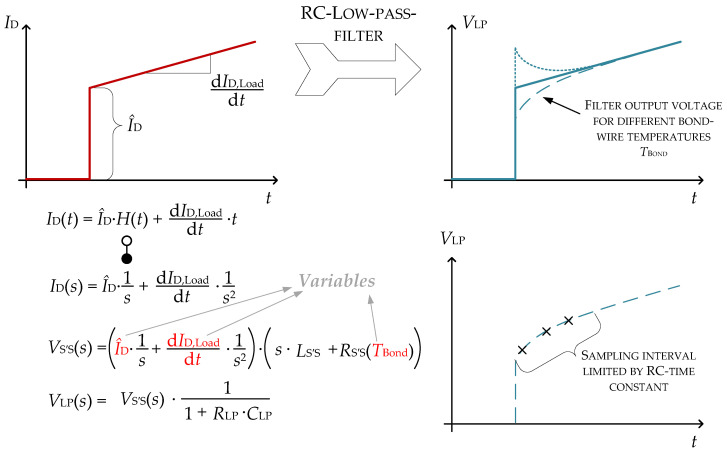
The signal used for measurement (low-pass filtered voltage *V*_LP_) is dependent on three variables, which can be shown quite easily in the Laplace domain. The resulting function can also be expressed in the time domain. If only one sample was taken after turn-on, it would not be possible to separate these variables. However, taking three samples provides a set of equations that includes these three variables, which may then be extracted by solving the set analytically or using an ANN.

The method can be enhanced to include more than two influencing parameters. One initial step may be to model a more realistic current shape than the ideal step function with a fixed step height $\hat{I}$ that has been until now. After the actual switching process, the current will ramp up or down according to the present load (see Figure 2). The resulting $di_{\mathrm{Load}}/dt$ is much smaller than the current slope during the switching process, yet it will significantly influence the sampling afterwards. Therefore, this parameter should also be taken into account, enhancing the method to three variables, for which a set of three samples is necessary (this has already been incorporated in a previous publication [24]).

To validate this approach, the method was tested using simulation and measurement data. For this purpose, a simple inverter phase was implemented in a test rig to enable the triple-sampling and the corresponding signal transmission presented. When operating at different temperatures, it is possible to clearly see the influence that this parameter has on the set of samples.

## 3. Application of Current Sensing

A corresponding triple sampling system was also successfully commissioned in inverter mode and performed very well at constant temperatures. Thanks to the system’s inherent oversampling, the individual sampling values achieved a very high level of accuracy, alongside robust sampling time accuracy. This resulted in deviations of ±1 A in the range under consideration.

**Figure 3:**
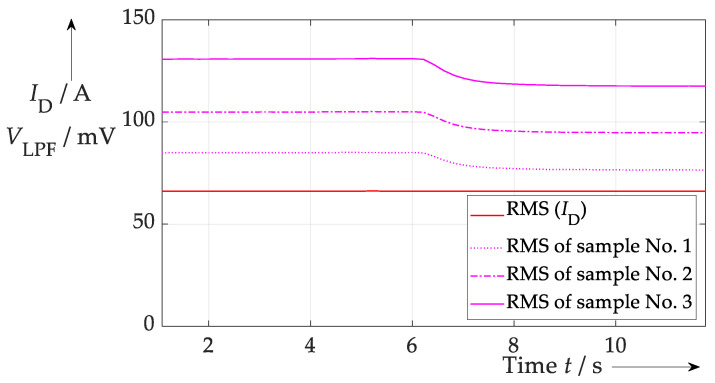
Test procedure for the temperature compensation in the proposed current sensing approach. At the beginning a higher temperature is present than at the end. Thus, all three samples show a higher RMS value first and then decline according to the temperature.

Figure 3 illustrates the difficulty of temperature compensation. It shows a measurement situation in which the current remains constant at one operating point, but the module is cooled down (more or less abruptly) between timestamps 5.0 s and 10.0 s. If only one sample from the integrator is used as current-sensitive signal, the current error would be approximately 11–12%, depending on which sample is used (first, second or last).

**Figure 4:**
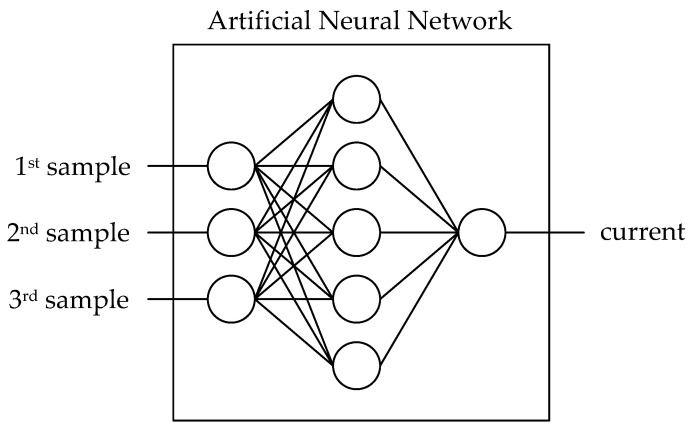
Basic structure of the current sensing ANN (schematic). Three samples are processed and are used to estimate the drain current.

As explained above, the compensation was then carried out using an ANN. To achieve this, training data generation was necessary. This was achieved using a procedure similar to that depicted in the above figure. For one current amplitude (one operating point) different temperatures were applied to the module. This involved self-heating the module due to losses without any cooling. External cooling was then abruptly applied at a certain point in time, leading to temperature variation. This procedure is a very limited approach to obtain the necessary current and temperature variations for a sensible training routine. However, it demonstrates how limited an approach can be in order to provide feasible training data. The time interval for the training data should then be placed around the temperature swing in order to obtain a wide range of training data. No additional precautions are required for the subsequent training, as this signal is sampled in the time domain. This means that a sine wave ranging from negative to positive values is sampled. Using this set of data, the training of the ANN is performed (see the scheme in Figure 4). The ANN consists of two hidden layers with seven neurons each, which are fully connected. It should be noted that the resulting shallow neural network is a simple feedforward network that uses activation functions such as tansig (for the hidden layers) and ReLU (as output layer). Training was performed using the Levenberg–Marquardt algorithm with a termination criterion of either a maximum of 6 validation failures or 1000 epochs. The damping parameter μ varied between 10^−3^ and 10^+10^. To ensure comparability, these parameters have been kept consistent across all the networks presented here and apply to all the subsequent results.

**Figure 5:**
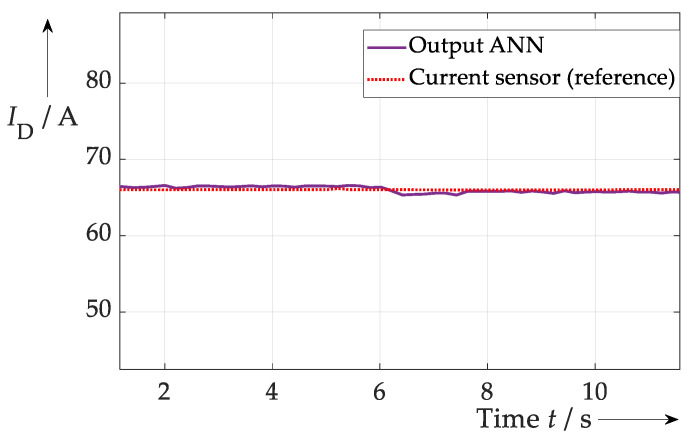
Result of current sensing during temperature swing at *t* ≈ 6.0 s. Due to a proper training of the ANN the temperature influence is mitigated. No significant change in current signal from ANN—purple—can be seen at *t* ≈ 6.0 s compared to reference sensor—dotted red.

If the three samples are used as input data for an ANN (with the current as targets) and a training routine is executed that includes the ranges of current and temperature used in subsequent tests, this error can be reduced to a minimum of approx. 1.0 A or 1.5% (see Figure 5). A small error still remains because, for example, the training data range among others was not optimised at that research stage. However, the test clearly shows that an ANN can effectively compensate the influence of the bond wire temperature on the presented current sensing method.

To obtain such a good performance, it is necessary to do some preprocessing of the input data of the neural network. The presented case showed that this was an effective way to filter the input samples. This meant that the ANN only needed to compare a quasi-ideal sine waveform of integrator samples to a quasi-ideal sine of current waveform. Otherwise, i.e., when noisy raw data is used, the current error is much higher than that depicted in Figure 5.

Clearly, higher-quality input data will lead to better results, which can be compared based on their performance, i.e., the remaining current error at different temperatures. But the question is: Does an ANN with a higher-quality input ‘only’ provide more accurate output values, or does it also learn fundamental correlations much better? The latter would be of utmost importance if only a limited range of input values are available. This is the case in many condition monitoring applications involving temperature variations. Extensive temperature variations and respective recordings are required, because a power module has to be heated in this case. However, if fewer operating points at different temperatures are required for effective training, this would constitute a significant advantage.

## 4. Explaining the Mechanisms of the ANN

Following this consideration, a method should be adopted that provides clear indications of the physical correctness of the trained network. More conventional artificial intelligence methods suggest well-established procedures, such as Hinton diagrams [25], or enhanced approaches, such as layer-wise relevance propagation (LRP) [26], to gain deeper insight into the black-box model of an ANN. However, these procedures focus more on assessing the inputs to the neural network. Ultimately, this means that it can be determined which inputs are relevant to the ANN and which are not. Other methods, such as calculating Shapley values or using LIME, focus more on the question of how the output is shaped by the inputs, and especially by how much. This may be useful, and it should be clarified in this paper, but it does not specify whether the ANN is based on underlying physics. Therefore, a simple yet unconventional approach was applied to this problem.

The idea behind this method is that an ANN with a physically consistent behaviour would exhibit some kind of characteristic curves corresponding to the underlying mechanisms. The challenge now is to make these characteristics visible to the user. This should be done in such a way that they can easily be assessed in terms of both their functionality and their accuracy. In addition, it should be easy to implement for the sake of simplicity. This sensitivity analysis of the neural network is a method that meets all requirements.

**Figure 6:**
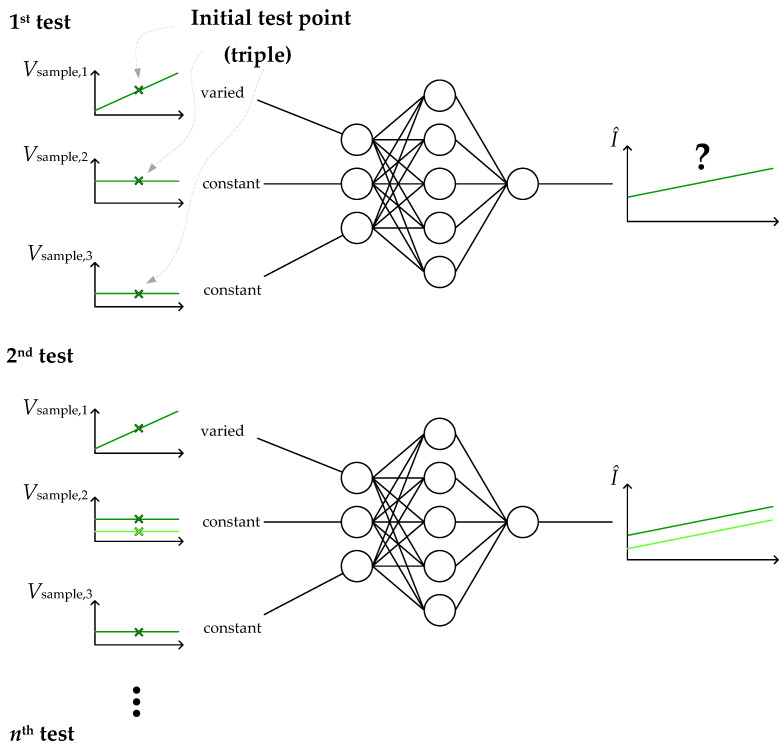
Proposed sensitivity test to gain more ‘explainability’ in the used ANN. Only one input of the ANN is varied (starting from a reasonable test point) and the output is plotted as a result (similar to ICE). To enhance the sensitivity test to another dimension (input), a second parameter is changed in subsequent steps.

Such a sensitivity test was performed with the ANNs by applying the following steps: Firstly, a training data sample is selected. One sample means one value per input signal. This sample should ideally lead to a reasonable value on the target side (i.e., no statistical outlier). To apply the aforementioned current sensing method, here three associated low pass filter values *V*_LP_(*t*_1_), *V*_LP_(*t*_2_), and *V*_LP_(*t*_3_) are used that lead to a sensible current value. The current value does not need to be taken from the training data set, because the ANN ideally should yield the correct output for a suitable sample anyway. This should be verified by feeding the three values to the input of the ANN. Then the actual test is performed: All inputs save one are maintained at a constant level, and one input is varied in a special manner. This variation could be implemented rather flexibly. For example, it could be ±10% of the initial value, or it could be distributed linearly or accumulated at interesting values across the whole range of occurring samples. As a first test, here a linear variation of ±10% around the initial value is presented and a schematic diagram of such a test is shown in Figure 6.

To provide more information about the state of the trained ANN, it is useful to show not only the variation of one single parameter but also to vary another parameter (at least by predefined discrete steps in order to preserve clarity). So, in the following plots the primary variation of inputs in the ANN can be seen at the *x*-axis of each plot. The different waveforms inside these plots, however, represent the gradual variation of a second parameter. The third (and, later in this paper, all additional) parameters remain at a reasonable level. This level is determined by the initially chosen test point: To obtain a viable test, it was necessary to define a set of inputs for the neural network that were within the range of the training data, or at least physically possible, beforehand. This test point is located at the very centre of the following plots, which means it is exactly halfway between the beginning and end of the line with respect to its *x*-axis value. It is also at the midpoint of the different waveforms (representing the second varied parameter).

Contrary to the mentioned explainability methods like ‘**L**ocal **I**nterpretable **M**odel-agnostic **E**xplanations’ (LIME) or ‘**Sh**apley **A**dditive Ex**p**lanations’ (SHAP), the proposed test routine is much easier to implement. However, its explainability is only increased to a limited extent. To distinguish the proposed method from conventional ones, the latter will be presented briefly.

It should be noted, however, that the sensitivity test presented here is only useful for an initial assessment of whether the neural network has actually learnt physical realities. A test that appears sensible at first glance does not necessarily correspond to a generalised, properly trained network. This is particularly important when the expected characteristic curves resulting from the sensitivity test are not actually known. If they are known, as demonstrated below in the current measurement, the sensitivity test can, of course, reflect the characteristic curves as a reference. Furthermore, if a test yields difficult-to-interpret results, a more detailed analysis may not be necessary, as it can be safely assumed in this instance that the network is highly specialised.

### 4.1. Differences to Conventional Methods of Explainability in AI

More elaborate approaches can be found in the aforementioned SHAP. This method focuses more on the inputs and how they contribute to the present target value. Compared to the proposed method, this essentially looks in the opposite direction through the ANN, because a—more or less—fixed set of inputs is considered here, rather than a fixed set of outputs. Nevertheless, the idea of the two approaches is similar: In both approaches the significance of inputs to the whole system can be measured. SHAP illustrates this very clearly in the respective plots. In contrast, the proposed method requires some interpretation of the outputs to reach the same conclusions: If the output in Figure 6 has a high slope, the varied input parameter will also have a high significance and will contribute substantially to the output. If, however, the resulting slope is very small or even zero, this means that the input (which was varied) will not contribute to the output at all. Let it be said that in the proposed sensitivity test the situation is only depicted for one input instance (set of samples). To gain a more holistic statement this test must, of course, be generalised. This must also be done using SHAP, as it is also a local explainability method (a globalisation of the mean SHAP values is, however, often done with mean Shapley values [27]).

Another local method of explainability can be found in LIME, e.g., applied in [28]. This approach is more similar to the proposed method, because it also focuses on the output at a variation of inputs. In contrast, the inputs are varied randomly, and the most interesting step of LIME is the (local) linearisation of the outputs, which is not performed in in the proposed method. Nevertheless, the idea is again comparable to the intention of the proposed explainability method, which is the assessment of outputs at varied inputs whether this forms reasonable waveforms or not. Both SHAP and LIME require a higher degree of explainability test, but the resulting application is ready to use with less effort required to interpret the test results. The presented method, however, is easier in using on trained ANNs but then requires more interpretation by the user. It is therefore more basic in structure.

It is therefore more comparable to also more basic conventional approaches like ‘Individual Conditional Expectation’ (ICE) and ‘Partial Dependence Plots’ (PDP). The proposed method is a local form of ICE, only considering a limited variation of two parameters. However, the procedure of the presented approach is very similar to ICE. If the variation was performed more broadly and more samples were used, the result would be a typical ICE plot. The aforementioned plots are typically used to gain insight into the significance of certain inputs to the outputs [29], a task that can also be accomplished using the presented plots. This paper instead focuses more on the quality of the resulting ANNs. This means the intention is to assess the different ANNs rather than the parameters used.

### 4.2. Evaluation of Trained ANNs with Sensitivity Test

As mentioned in the previous paragraph, the application of explainability methods in this paper focuses on the quality of the trained neural network. Therefore, different training situations were performed which differed, for instance, in the training data provided. Initially, it was expected that high-quality training data (very accurate, smooth, etc.) would lead to a better performance than poor data (e.g., very noisy). However, the question remains as to whether this is true, not only for the performance of the ANN, but also for the degree of physical understanding of the ANN (where some explainability is needed).

**Figure 7:**
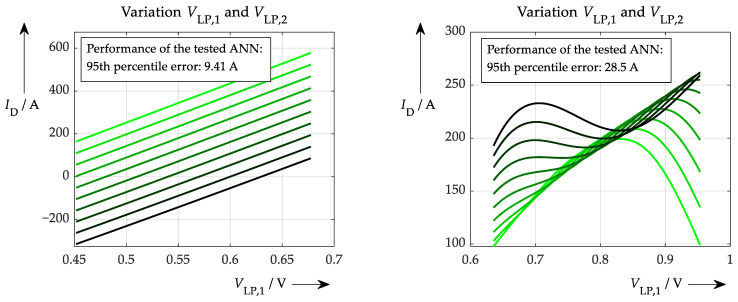
(**on the left**) Proposed sensitivity test applied to a well-trained ANN, which was fed with smooth input data. (**on the right**) The different lines represent different values for the second sampled voltage (*V*_LP,2_). The same test conducted with an ANN that was trained with high-noise data and therefore showed a worse learning procedure.

Figure 7 shows the result of the explainability test for two different situations. On the left, a proper training of the ANN had been performed. This means that the training data were very precise and not affected by any noise. By contrast, the right-hand plot in Figure 7 shows the result of the test routine following poor learning behaviour, where heavily noisy input data were used. To get more information out of the test routine, not only was the *V*_LP1_ parameter varied (seen on the *x*-axis), but also the second sampling value *V*_LP2_ was varied gradually (resulting in colour-coded waveforms from black to light green). However, the main result can be seen very clearly also in one single line if compared to the respective other situation. Where the good training results in a straight ascending line during increasing *V*_LP1_ value, the poor training shows a very inconsistent relationship between sample value and output of the neural network.

Now, the test results should be examined in more detail to determine the strengths and weaknesses of this method of adding explainability. Firstly, it is obvious that the training with suitable training data produces to a highly structured output leading to the assumption that the ANN has learned some correlation between input data and targets and that this relationship is also quite linear. This already provides an initial indication that the neural network may indeed have learnt physical relationships. If the detailed set of equations underlying the respective problem is not known before applying an ANN to it—otherwise these correlations could be used to solve the problem more accurately—it is sensible to prioritise the left ANN in Figure 7 over the right one, not only because of its superior performance, but also because of its more plausible explainability. However, if the exact equations are not known, but the dependencies can be roughly assumed, this method will also be helpful. For, example, in a hypothetical case where some inputs and targets should show a quadratic correlation, a similar curve must be the result of this quick explainability test. Any other waveforms would indicate an incomplete understanding of the physics by the ANN.

**Figure 8:**
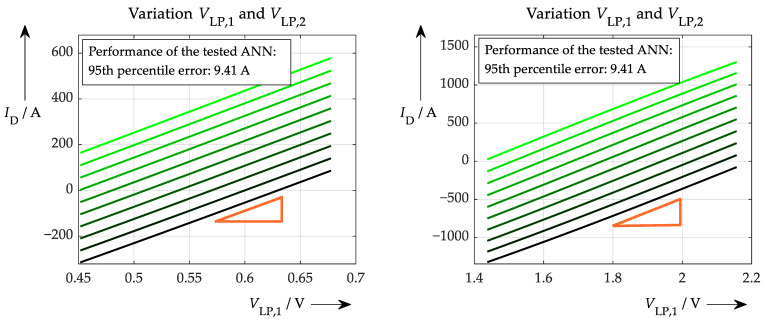
Applying the test routine to a well-trained ANN at two different test points. While the results are different in absolute numbers, the slope (orange triangle) is very similar.

The main drawback of the explainability method presented is that it is a very localised test because it uses only one set of input data. To generalise the findings, this process has to be repeated for multiple test points, as shown in Figure 8 (at least for another one). However, because of the simplicity of the test, this can easily be done with additional data. Figure 8 shows another ‘good’ example. This is to expand the scope of the previous test and obtain further evidence of improved learning behaviour, i.e., physics-based or at least consistent.

The resulting correlation is very similar to the first test point. However, the data sets are completely different in the two cases. The first sample shows a filter output voltage of approx. 0.57 V on the left and 1.8 V on the right. The absolute dependence of the current regarding the actual filter voltage is approx. 1740 A/V on the left, and slightly less at 1710 A/V on the right. Taking into account the similar waveform and the resulting slope of these two completely different test points, it can be concluded that a well-trained ANN is present.

**Figure 9:**
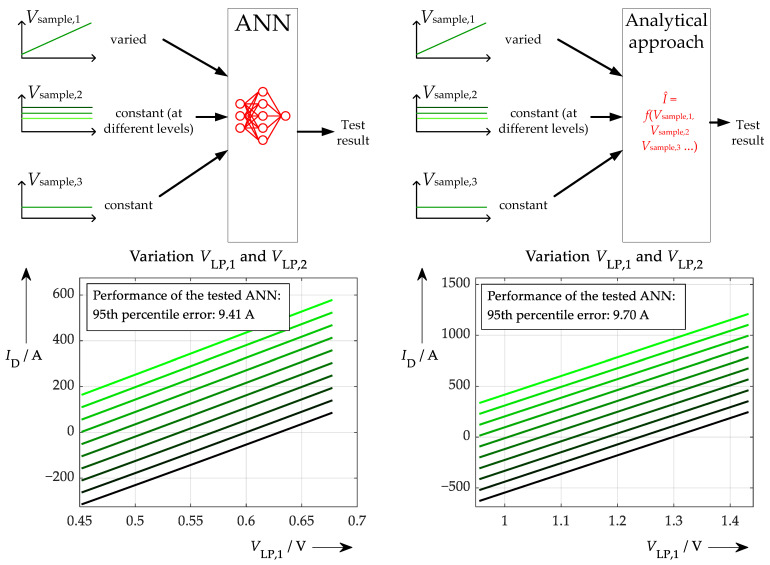
(**top on the left**): Sensitivity test of a well-trained ANN at a certain test point compared to (**top on the right**) the test routine applied to the formulae for re-calculation at the same test point, and (**bottom**): the respective results (**on the left**) using the ANN, (**on the right**) for re-calculation.

Determining the current from a set of three samples derived from a current pulse and subsequently processing it with a low-pass filter is a task that may also be calculated analytically, or at least with a simpler fit function. This approach may not be that efficient in terms of calculating time compared to a neural network and it requires more prior knowledge, but it should in turn demonstrate a very high accuracy. In the actual, rather simple task involving three voltage samples and the corresponding current, analytical equations were used. Therefore, it was necessary to solve a set of equations like Equation (6) analytically (each with the respective sample time inserted) for the current $\hat{I}$. Such a solution was found and implemented as an alternative path to the ANN-based approach (it achieved the same level of accuracy as the well-trained ANN). The same explainability test was conducted with this solution (keeping two parameters constant and varying only one ‘input’). A direct comparison can now be made between the sensitivity results for the ANN and the analytical approach, as shown in Figure 9.

The two methods produce similar output, suggesting that both the ANN and the analytical approach perform well and also follow the same underlying equations. However, it must be made clear at this point that the proposed sensitivity test itself—and thus the explainability approach—must not be overinterpreted, because it may vary parameters that do not occur in real applications. To stick to the presented problem, the first sample value of the filtered waveform will never increase by +20% while the other two samples remain the same. This cannot occur due to a higher current step (here all samples would show a higher value) nor due to a higher temperature (in this case, it is more likely that the values of the second and third samples would change more than the value of the first sample), or due to another d*i*_Load_/d*t* being present (a similar effect to the temperature difference). Therefore, only limited insights can be gained from these presented examples of sensitivity tests. Nevertheless, a more sensible test could be designed in terms of physical correctness. For example, the third sample could be varied, with the second sample varied step-by-step to create a test routine representing a variation in temperature or d*i*_Load_/d*t*. In this case, however, the influence of temperature or d*i*_Load_/d*t* would need to be known in order to compare this correlation with the results of the sensitivity test.

## 5. Enhancement to Multi-Parameter Sensing

Until now, the task for the ANN was a quite straightforward, as it only calculated one parameter (drain current) from the three samples of the low-pass filtered signal. This could also be achieved by using an analytical approach. However, the sensitivity test should also be applied to a more complex structure of parameter extraction. To achieve this, the current should not be the only target and the inputs should consist of more than one signal (filter output signal, three times sampled). To obtain more information from of the power semiconductor module, additional signals may be measured; in this study these are the on-state voltage of the semiconductor and its turn-on delay time. The on-state voltage provides information on the junction temperature (not to be confused with the overall bond wire temperature used until now!), but also depends on the load current [30,31] and the turn-on delay time solely depends on the junction temperature of the power semiconductor [32].

### 5.1. On-State Voltage Measurement

There are various methods for measuring the on-state voltage of the power semiconductor, with their own advantages and disadvantages. While good-performing solutions with an additional power semiconductor that is ‘normally on’ [33] may work well, they exceed the constraints of a low-cost solution. Furthermore, additional MOSFETs may be more difficult to control. A good trade-off between complexity and accuracy was found in a solution using two decoupling diodes [34] that provide a low maximum voltage but make the on-state voltage accessible.

**Figure 10:**
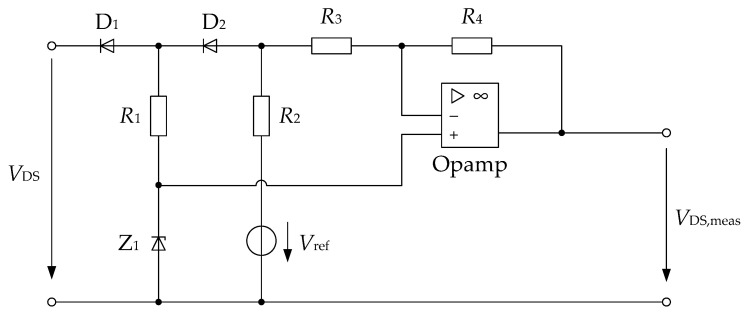
Decoupling method for the *V*_DS,on_-voltage of the semiconductor switch (not depicted) at low voltages (switch conducts) according to [34].

This voltage decoupling method is depicted in Figure 10. This method was tested as a reference measurement method for this parameter and demonstrated a good accuracy of ±1% at higher voltages and up to ±5% at low voltages. This is consistent with the original publication of this method, which states an overall accuracy of ±2% [34]. Thus, this part of the signal acquisition can be considered fairly accurate and can therefore be used for the ANN-based processing to gain information about junction temperature and to strengthen the confidence in drain current determination (because the current measurement is already performed using the three-sample method). A drawback of using this signal is the fact that the on-state voltage depends on more than one parameter (mainly the load current and the junction temperature) and that the correlations between the parameters and the signal may not be linear. The proposed enhanced ANN is therefore intended to separate the different underlying effects.

### 5.2. Turn-On Delay Measurement

However, determining the turn-on delay of the semiconductor switch is a more difficult measuring task in view of accuracy. This parameter is a valuable one because it is not susceptible to other influences and therefore provides qualitatively good information about the semiconductor junction temperature. On the contrary, however, the sensitivity between the measured signal (the difference in delay time) and the value to be determined is very small, adding up to approx. 2.0 ns per Kelvin temperature difference [32], but only for an IGBT with a very long overall turn-on time. In test measurements with faster switching SiC-MOSFET, the authors found a much lower sensitivity of only one tenth (approx. 0.18 ns/K with Infineon’s FS03MR12A6MA1B (obtained at the author’s location), switched with a gate resistor of 4.7 Ω, which is slightly lower than the rated external gate resistor of 5.1 Ω). The measurement setup was similar to that in [35] and consisted of a trigger activated by the gate control voltage and another trigger detecting a current slope by means of the parasitic inductance of the power module. These two triggers themselves were inputs for a time-to-digital converter.

Other publications also imply lower sensitivities for SiC-MOSFETs. For example [35], derives a sensitivity of 0.8 ns/K in a special switching process that starts with a resistor of 2000 Ω to prolong the turn-on time, followed by actual switching with a resistor of 10 Ω. Nevertheless, the sensitivity is very small, and a considerable effort is required to achieve acceptable accuracy with this measurement signal. Even when using fast time-to-digital converters for recording (in our own measurements we used a commercially available Texas Instruments TDC7200, obtained at the author’s location), a scatter of ±0.7 ns had to be most of the time in various double pulse tests after reasonable averaging of 20 consecutive pulses.

## 6. Combining All Disposable Signals into One Sensing System

**Figure 11:**
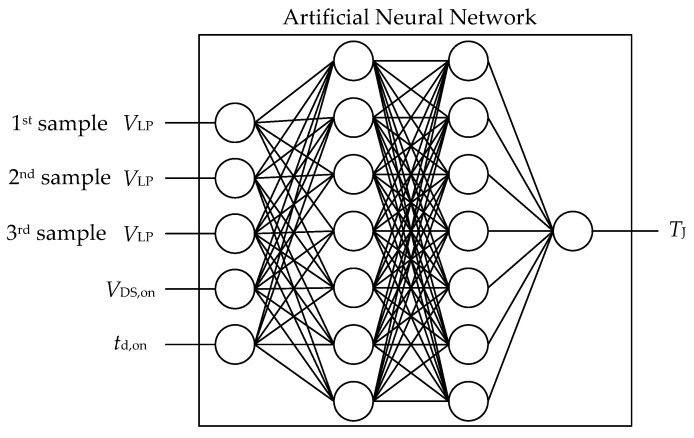
Schematic structure of an ANN for temperature estimation. The temperature information is derived from the on-state voltage and the delay time. The on-state-voltage, however, depends on the drain current, which can be determined using the parasitics-based low-pass voltage.

However, the overall accuracy of the turn-on delay time, even though it is quite poor, is not of utmost importance in this setup, because it is the only signal containing temperature information. What is interesting is how the signal, which is difficult to obtain accurately, can be combined with a signal that is easier to measure (*V*_DS,on_), which, however, depends on different parameters. To simplify the situation, the following network, consisting of all possible inputs and providing only the junction temperature, will be considered. This network structure is shown in Figure 11.

Such an ANN should now be trained and tested using the proposed explainability method. To gain a broader insight into the question of how accurately the signals are expected to be measured, the training and subsequent testing were conducted using simulated data and an additional simulated level of accuracy. There are several reasons for initially using simulation data. Firstly, it enables us to create an ideal scenario with predictable outcomes and easily interpretable sensitivity tests. Secondly, it makes it relatively straightforward to adjust the error level with which a particular signal was measured, allowing more flexible modelling of the measurement. Thirdly, it enables us to exclude any cross-correlations that are not to be addressed in this paper for the time being. The simulation was carried out using current values derived from analysing of the composition of the various input parameters (see above). Mathematical formulas could simply be applied for this purpose, as was also the case with the turn-on delay time. For the forward voltage of the semiconductors, a corresponding output characteristic and a simulation of a switching circuit had to be set up. However, as it is not possible to simulate every possible source of disturbance, the processing of measurement data will also be covered later.

**Figure 12:**
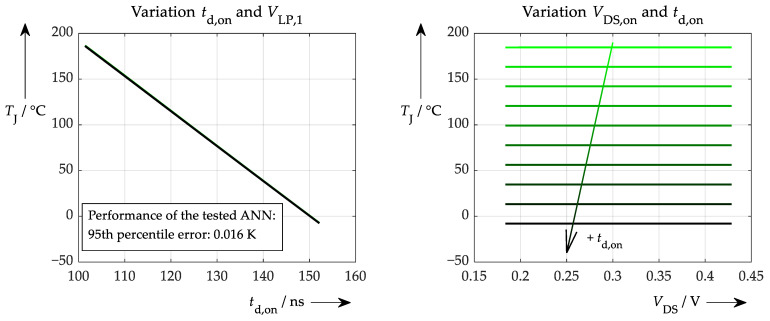
Explainability test with an ANN trained with ideal, simulated data. (**left**): Variation of the turn-on delay time (actually for various *V*_LP,1_ values, which, however, result in curves that overlay exactly). (**right**): Variation of the on-state voltage (has almost no influence on the resulting temperature) for different turn-on delay times. Different colours represent rising turn-on delay from green to black.

The first situation is a scenario in which only simulated and exact data were used for training and testing. Such an ANN performs ideally and achieves 100% accuracy. More interesting, though, is where the ANN gets its information from (which signal, to what extent, etc.) and whether conclusions can be drawn regarding the physical correctness of the network. These questions can be answered by applying the proposed sensitivity test (Figure 12).

In this case, the influence of *t*_d,on_ is dominant, as can be seen in Figure 12 on the left. Varying this parameter over its full range results in a full range of temperatures. The variation of the second parameter (values for *V*_LP,1_) has no effect whatsoever. Additionally, no temperature information is extracted from the on-state voltage; only horizontal curves are obtained from the explainability test, as can be seen on the right-hand side of Figure 12. Once again, the secondary variation of *t*_d,on_ is solely responsible for the temperature output of the ANN. This result was expected and can be interpreted straightforwardly: all of the ANN’s information comes from the turn-on delay time. The next step is to answer the question of how the situation changes when non-ideal simulated data are used for training and testing with a certain amount of noise added beforehand to represent the measurement inaccuracy of the respective signals. These accuracies are summarised in Table 1 below.

**Figure 13:**
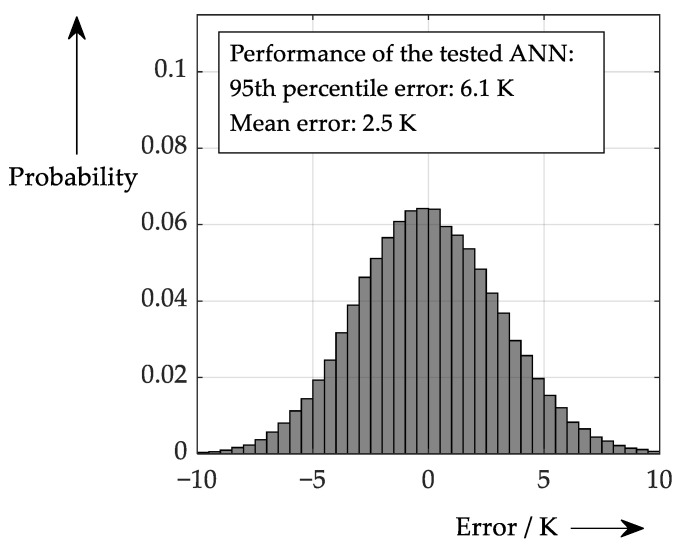
Performance of ANN processing data with limited accuracy (see Table 1).

To make this as realistic as possible, the accuracies determined in previous chapters are applied. In this case the performance is worse than in the initial test, which can be seen in Figure 13.

**Figure 14:**
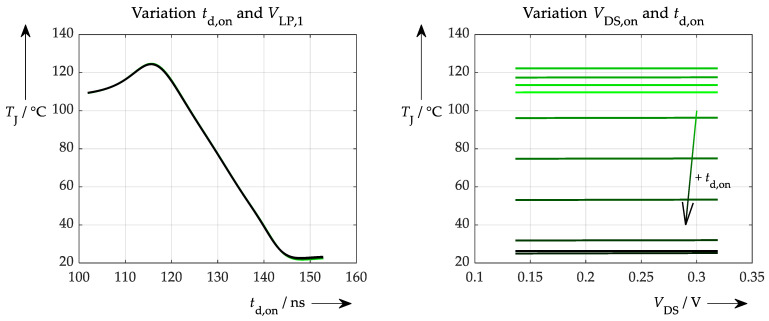
(same procedure as in Figure 12, but with different ANN): Explainability test with an ANN trained with more realistic, simulated data. (**left**): Variation of the turn-on delay time (actually for various *V*_LP,1_ values, which, however, result in curves that again overlap exactly). (**right**): Variation of the on-state voltage (nearly no influence on the resulting temperature) for different turn-on delay times (there is no longer a colour gradient across the entire plot, but only in sections; see arrow).

Apart from the poorer performance of the ANN, the degree to which physical correlations are learned may also have changed (at least insofar as these can be identified by the sensitivity test). This can be examined using the proposed explainability test, which is analogous to the procedures in Figure 12 and results in Figure 14.

As in the previous test, most of the temperature information is still drawn from the turn-on delay time. However, it is clear that the correlation between this parameter and the target value is not learned as well as without any accuracy limits (due to the quite inconsistent characteristic curve after approx. 140 ns in the left-hand plot). Furthermore, the secondary variation of *t*_d,on_ in the right-hand plot is no longer arranged from negative for lower temperatures to positive for higher temperatures. Instead, there are outlying curves amongst the others. This suggests that the ANN struggles to learn the physical relationships properly when provided with realistic data.

The next step will reveal whether a further deterioration in the accuracy of *t*_d,on_ influences the ANN’s preferred source of information. This was achieved by manipulating the accuracy ranges of the realistic approach in Table 1 as follows: The accuracy of *t*_d,on_ was worsened by a factor of two, while at the same time the accuracy of *V*_DS,on_ was halved simultaneously. The sampling of the low-pass filter voltage remained constant. This results in the new Table 2.

**Figure 15:**
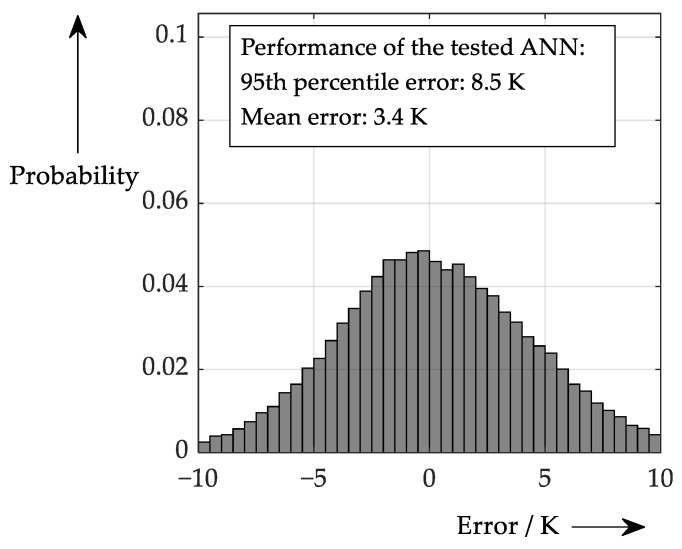
Performance considering a theoretical scenario with the accuracy values of Table 2.

Applying these accuracy ranges leads now to the performance in Figure 15.

**Figure 16:**
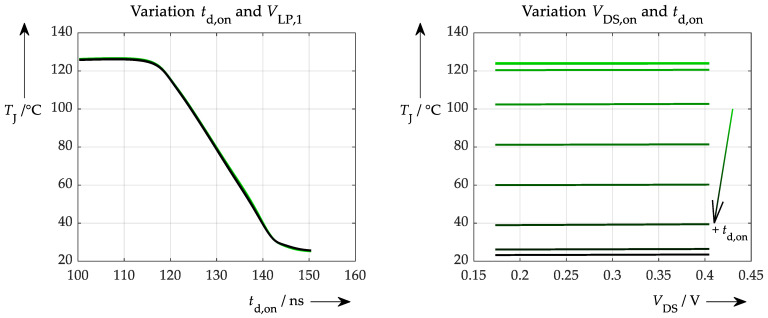
Explainability test in the hypothetical situation of less accurate turn-on delay measurement and more accurate on-state drain-source voltage determination. (**left**): Variation of the turn-on delay time (actually for various *V*_LP,1_ values, which, however, result in curves that overlay exactly). (**right**): Variation of the on-state voltage (nearly no influence on the resulting temperature) for different turn-on delay times.

Performance is now worse in both a broader distribution and a slightly asymmetric distribution. This may be due to an inferior underlying trained model, which can easily be investigated with the proposed explainability test. This was conducted in the same way as before, producing the results shown in Figure 16.

Interestingly, even though the ANN learns incorrect correlations, especially at very low and at higher values for the turn-on delay time, it still seems to draw its temperature information mainly from this parameter. Nevertheless, the low-pass filter voltage has no significant influence on the junction temperature (waveforms overlay each other in the left plot), which is correct. Another insight is observed in the right-hand plot (when compared to the previous plots of the same procedure), namely that the waveforms are no longer exactly horizontal. This suggests that the ANN is now gaining information from this parameter as well. Admittedly, this influence is very small: <10% at a variation of ±50% of the initial value for *V*_DS,on_.

**Figure 17:**
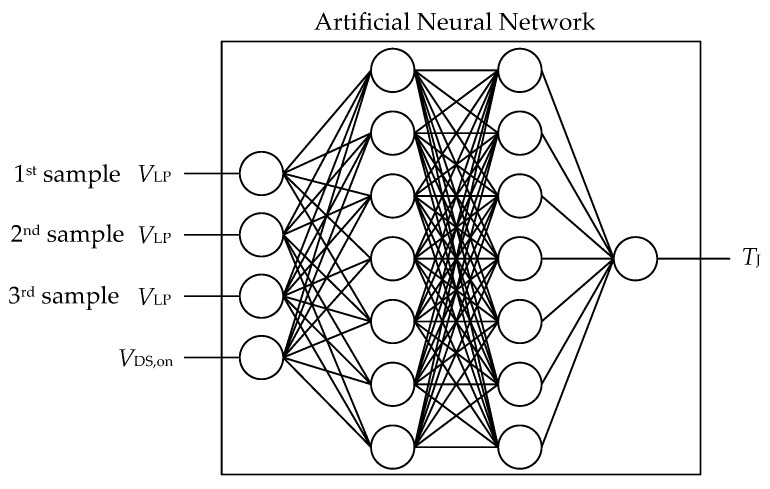
Schematic structure of an ANN for temperature estimation. Temperature information is derived solely from the on-state voltage. It is, however, dependent on the drain current, which is determined by the parasitics-based low-pass voltage.

Even a poor *t*_d,on_-measurement does not appear to lead to a neural network that prefers a different parameter for information gain. This is understandable, because *t*_d,on_ is a very good signal to accurately determine the junction temperature compared to the multiply influenced on-state voltage. Therefore, if only the junction temperature needs to be determined and a more or less accurate *t*_d,on_-measurement is available, it is not necessary to use an ANN. However, if the current is also of interest an additional signal must be considered. Moreover, the situation is different if the turn-on delay time is unavailable. This case will be considered alongside with the previously presented procedures. The ANN then looks as shown in Figure 17.

**Figure 18:**
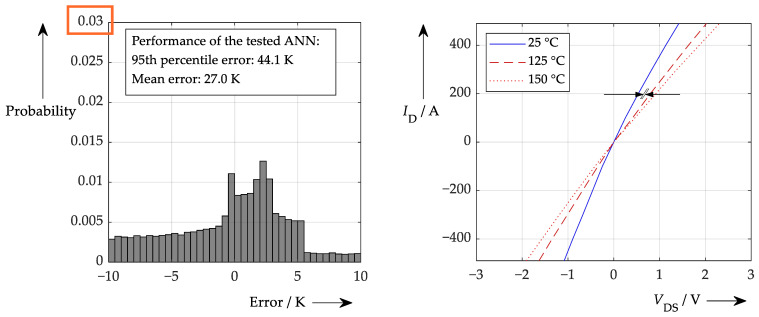
(**left**): Performance of an ANN like that shown in Figure 17 with realistic error values applied (note the maximum probability 0.012). Problem is the high sensitivity of the on-state voltage to the temperature according to the output characteristic shown on the (**right**) (for three temperatures).

Applying the accuracy values determined according to Table 1 leads to the following performance of Figure 18. This shows that, in this case, it is not possible to make a reliable temperature calculation.

**Figure 19:**
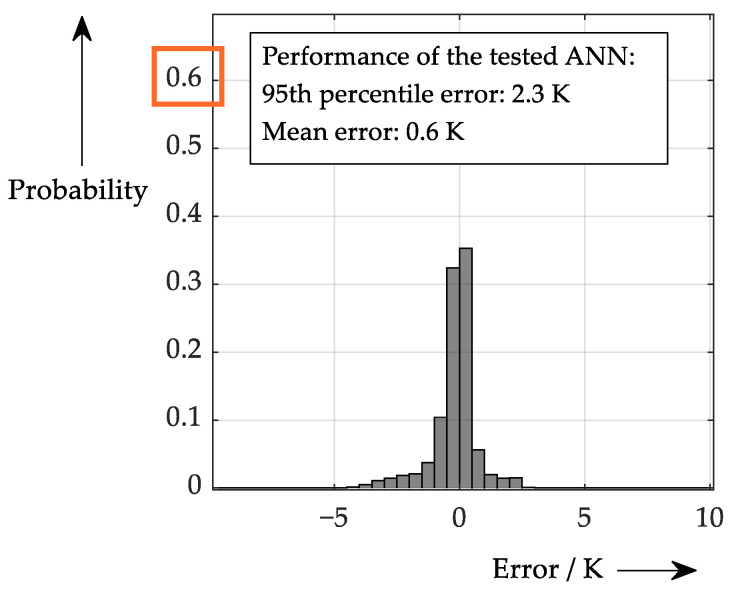
Performance of a procedure similar to that in Figure 17 but with ideal simulated data for both training and testing (note the probability scale).

As this procedure did not yield any sensible results, any further investigation of this situation was omitted. However, the reason for this poor performance is not primarily due to the ANN or the application of AI itself but rather stems from the underlying problem that needs to be solved. If the on-state voltage is the only component responsible for providing the temperature information, this parameter has to be determined very accurately. Yet, in this study, an overall accuracy of just 1.7% of the full scale is applied. This means that, especially at low currents (and thus low on-state voltages), an even lower level of accuracy is achieved. According to the output characteristic of the used semiconductors (shown in Figure 18, right), at a representative test point near *I*_D_ = 200 A, a variation of 26 mV in the measured on-state voltage would yield a junction temperature deviation of approx. 10 K. At lower on-state voltages this number even increases and makes temperature sensing very difficult, not to mention learning from such data. In this case, it may be better to consider the output characteristic in advance and to try to apply some kind of data smoothing. However, if smoothed data are available, reasonable learning is also possible by the ANN, as shown in Figure 19, where ideal simulated data rather than noisy data were used for training.

**Figure 20:**
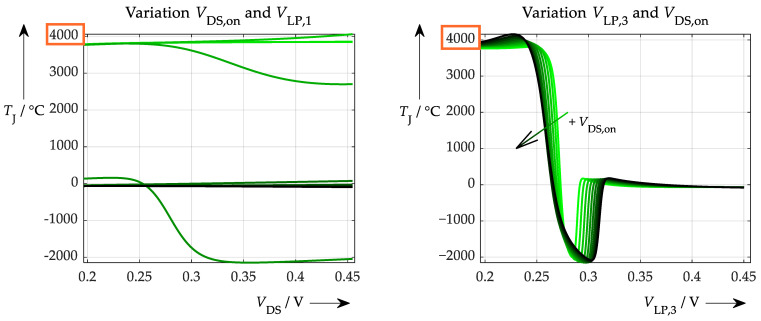
Explainability test for ANN that processes on-state drain-source voltage and the threefold sampling. (**left**): Variation of the on-state voltage at different values for *V*_LP,1_. (**right**): Variation of the third sample value at different on-state voltages (note that this scaling only makes sense in theory).

This procedure does not yield 100% accuracy (unlike in the idealised case of the triple sampling), because no meaningful temperature estimate can be derived at zero load current (see also Figure 18 on the right). Since such cases were included in the performance test, some larger errors are always present. Given this result, it is also possible to conduct an explainability test as shown in Figure 20.

**Figure 21:**
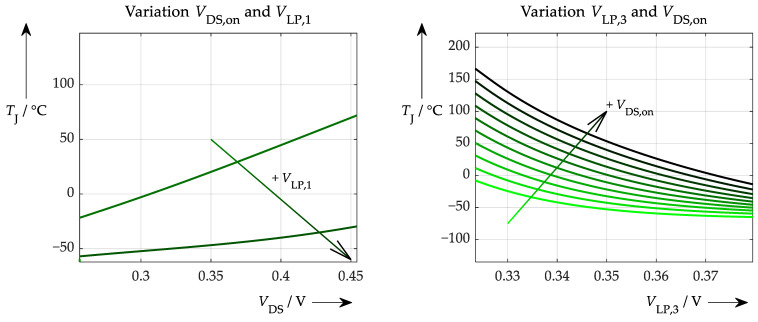
Detail of Figure 20: Explainability test for ANN processing on-state drain-source voltage and the threefold sampling. (**left**): Variation of the on-state voltage at different values for *V*_LP,1_. (**right**): Variation of the third sample value at different on-state voltages (colours now represent either the first sample value or the forward voltage value, respectively).

Even in the most ideal scenario, looking into the ‘black box’ does not produce a ‘smooth’ picture; rather, it produces an inconclusive picture. However, the results of the explainability test range from very low to very high outputs, neither of which is realistic. This behaviour may be due to problematic inputs (*V*_DS,on_ near zero) or the fact that sensible learning is difficult in this scenario. To gain a better understanding of this issue, Figure 21 provides details of the range of sensible output temperatures.

The detail shows reasonable values for the ANN output (junction temperature) and a smaller input range for the main varied parameter (*x*-axis). Here, a more coherent picture emerges. On the left, where the on-state voltage is varied, only one line representing one value for the first sample (*V*_LP,1_) remains. This sensitivity seems quite plausible for a single specified current (assuming that *V*_LP,1_ mainly depends on the drain current). Sensible data can also be derived on the right. Assuming that the main varied parameter, *V*_LP,3_, is again assumed to be significantly dependent on the drain current, it can be concluded that the ANN deals with higher currents on the right-hand side of the plot. The different lines represent various on-state voltages, meaning that if a certain *V*_DS,on_ is detected at higher currents, a lower temperature must be present. In fact, for the same current, the *V*_DS,on_ is higher at warmer temperatures (see also Figure 18). In conclusion, it is also possible to verify this at first glance inconclusive behaviour.

### 6.1. Applying the Explainability Test to Measurement Data

A sensitivity test in the above-mentioned manner can also be conducted using real measurement data instead of simulated data alone. When interpreting the results, it is important to consider the following possible effects. As measurement data, by its nature, will not cover all possible occurring operating points, but rather a selection of them, the ANN will often become specialised to the available data, making good generalisation more difficult to achieve. Thus, the t sensitivity test assessment must be adjusted according to the amount of available (training) data. Another very important factor is the quality of the available data and how the raw data is processed before it is fed to an ANN for training purposes. As demonstrated in the previous sections, smoothing the available data produces a much better ANN, i.e., one with better generalisation, than using raw data or at least insufficient processing of the available measurement values.

**Figure 22:**
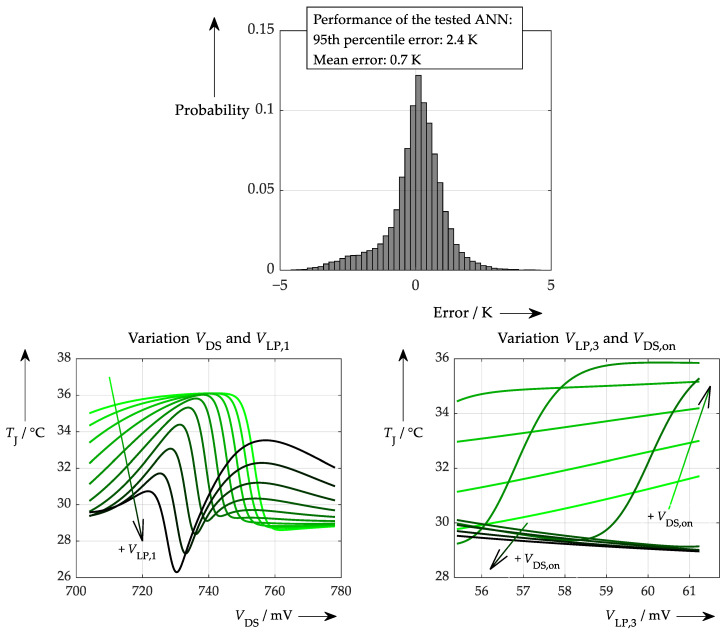
Despite showing a narrow and centred error distribution and thus an acceptable performance (**top**), the sensitivity test shows a more inconsistent behaviour, typical for non-generalised ANNs (**bottom**). This test was performed with a much smaller range of variation in the input data (be it in the first variable, as well as in the second variable).

Thus, the first experiment using measurement data should consider a situation in which the data was highly smoothed and only a small section of the whole range of junction temperatures was considered. This circumstance is further reinforced by the restriction that in a typical setup, the junction temperature cannot be adjusted (or better: determined) independently of the operating point (for instance, because it is determined by the load current) and the module temperature (which, in turn, affects the bond wire temperature). The test rig used consisted of a classical half bridge with an external heating option, which introduced some (limited) degree of temperature variation independent of any load condition. To generate training data, a specific operating point was selected and maintained for a sufficient period to ensure homogeneous heat distribution, thereby ensuring consistent bond wire and junction temperatures. The signals were sampled every 120 µs, resulting in a dataset of roughly 0.83 M samples over 100 s. Additional cooling was then applied to enable different temperatures at the same periodically changing load currents to be measured and stored as training data. This data was processed as previously described by smoothing to eliminate every noisy measurement uncertainty. For instance, smoothing was performed using a Savitzky–Golay filter with a window length of 40 ms until the on-state voltage and its complementary load current exhibited ideal characteristics as specified in the datasheet. This was applied to a 2.0 Hz test signal. On average, this corresponds to 333 consecutive switching events, which can be considered the maximum possible smoothing. This is valid for training purposes at least. During operation, a smaller time span may be applied to the smoothing window. The main smoothing can then be done using temperature values that do not change rapidly. These still time-dependent data were then used as training data for an ANN similar to the one used in the previous simulation experiments. The result of the performance test and the subsequent sensitivity test can be seen in Figure 22 below.

Considering these results, some very interesting conclusions can be drawn. At first glance, the performance (which provides error values for the test points as the distance between the ‘answer of the ANN’ and the ‘right answer stored beforehand for this test point’) is quite good, especially compared to the much worse values obtained for all-simulated data (see Figure 19). However—and this is the key point of this entire explainability assessment—this does not necessarily mean that the ANN has learned the physics behind this experiment; it may simply have stuck to some levelling of the overall provided training data. This is clearly evident from the fact that sensible correlations of parameters can only be seen when the sensitivity testing range is heavily limited (i.e., a pattern is only recognisable in the limited view of Figure 22; note the small range of values). Moreover, this problem could be identified using the easy-to-use sensitivity test described above.

### 6.2. Comparing to Simple Fit Method

**Figure 23:**
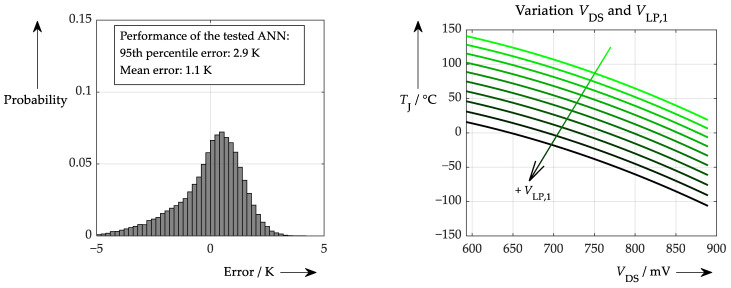
Performance (**left**) and sensitivity test (**right**) of a simple fit function consisting of quadratic and linear terms for every ‘‘input” (low pass filter samples and on-state voltage).

As a baseline approach, a second method is presented alongside the ANN, which can also be used to solve the problem of temperature determination. This method involves a simple fit of the input data to the output data, whereby in this case both quadratic and linear terms were selected for each input parameter. This does not necessarily have to correspond to physical reality; it simply needs to produce a plausible result. Using an automated fitting function, the exact data used for training the neural network was then employed. This resulted in an error distribution as shown in Figure 23 on the left.

In this case, a slightly worse performance can be observed compared to the approach using the ANN, which is not unexpected when a random fit function is applied. However, this fit function can be clearly reproduced by performing the proposed sensitivity test (see Figure 23 on the right). Though only one example varying two parameters is shown here, others would demonstrate the same correlations due to the fit function’s structure.

## 7. Discussion

At this stage, the results still need to be contextualised and discussed before a conclusion can be drawn. Firstly, the problem (current and junction temperature measurement) was examined in detail, and very cost-effective methods were found for each. While the use of simple neural networks worked very well for current measurement, further research was required for junction temperature measurement in order to obtain a meaningful result.

A simple, robust test was also presented to demonstrate how the resulting neural networks can easily be examined for underlying mechanisms. However, it would be more advantageous if such mechanisms, or better still the underlying physical correlations, could be integrated from the start. The disadvantage of this approach is that the necessary correlations must first be identified. In such a case, however, mathematical calculation would once again be conceivable, and neural networks would no longer be advantageous per se. A good middle ground is offered by methods such as those presented in [36]: here, rather than storing complete mathematical relationships, individual aspects of the network training are modified with specific adjustments so as to result in a better network, or at least one that is grounded in physics.

Another aspect that has to be addressed regarding the matter of current sensing and junction temperature is safety. In this context, however, it is important to distinguish between current measurement, which must be highly accurate and available rapidly, and junction temperature measurement, which is used for condition monitoring, for example, and therefore has lower requirements, particularly in terms of measurement speed. In principle, the method described here provides information on both current and junction temperature at the end of each switching operation. While this makes temperature measurement particularly fast, however, this advantage is somewhat reduced by the need to average the data to improve accuracy. Nevertheless, when measuring current in particular, it is possible to exploit the fact that the voltage is initially captured via parasitic inductances inside the module. This can be used for a parallel-connected short-circuit detection, for example.

## 8. Conclusions

The findings of this study are twofold. Firstly, it was demonstrated that the current could be determined very accurately by processing the voltage across the module’s parasitic elements using a simple ANN. Using this approach, the need for an additional module temperature sensor was eliminated—such a sensor would not have been helpful anyway, as detecting the correct temperature is nearly impossible—by only sampling the signal multiple times. When using this approach for current sensing, factors to consider are the possible variation in parasitic resistances and (less) parasitic inductances from one module to another and the crucial task of timing all samples exactly. The latter requirement was successfully demonstrated using an FPGA controller during inverter operation.

The second subject area involves a more detailed investigation of the used ANN itself and its enhancement with more input data and, in turn, the more challenging task of determination of the junction temperature of a power semiconductor module. It is important to find an easy method to gain insight into the usual black-box model of the ANN. A method has been found that is very similar to the Individual Conditional Expectation (ICE) method, but which varies two parameters simultaneously (rather than more instances at the same time). This method is easy to apply and requires limited effort to interpret the test results. Ultimately, the question of whether the ANN has learned characteristic sensitivities (which already suggests that an underlying physical correlation has been learned more effectively) can be derived from the test outcome. This is more difficult, however, when the network is poorly trained. In such cases, the resulting plots are difficult to interpret and only allow the conclusion that the training was poor, offering hardly any deeper insight.

Another finding of the study is that determining the junction temperature using the proposed current sensing method and of the semiconductor’s on-state voltage (via its output characteristic) is quite difficult and not particularly advantageous when using an ANN. Even when it is assumed to be much more inaccurate than it actually is, the additional (or in lieu thereof) measurement of the turn-on delay time significantly improves the accuracy of the junction temperature. In this case, it was shown that the main temperature information is always drawn from the turn-on delay time.

In summary, this paper makes a distinctive contribution to the assessment of alternative (i.e., sensor-less) current and temperature sensing in power modules, especially with regard to the evaluation of available signals with the help of artificial intelligence, and to the important and emerging challenge of explaining the neural networks achieved.

## 9. Patents

The section concerning current sensing with threefold sampling of a low-pass filtered voltage tapped across source and auxiliary source is patented under DE102021211410.

## Figures and Tables

**Table 1:** Applied accuracies to the respective signals.

	*V*_LP_-Measurement	*V*_DS,on_-Measurement	*t*_d,on_-Measurement ^1^
Accuracy (absolute)	1.4 mV	26 mV	0.7 ns
Accuracy (percentage of full scale)	0.13%	1.7%	1.0%

^1^ mean value for 20 consecutive pulses. Averaging these values is permissible because it is independent of the load current and the junction temperature changes much slower.

**Table 2:** Applied accuracies to the respective signals for manipulated test.

	*V*_LP_-Measurement	*V*_DS,on_-Measurement	*t*_d,on_-Measurement
Accuracy (percentage of full scale)	0.13%	½ ∙ 1.7% = 0.85%	2 ∙ 1.0% = 2.0%

## Data Availability

The raw data supporting the conclusions of this article will be made available by the authors on request.

## Abbreviations

The following abbreviations are used in this manuscript:

ANN	Artificial neural network
LP	Low-pass (filter)
DS,on	Drain-source during on-state of semiconductor
d,on	Turn-on-delay
